# Non-pharmacological Treatment for Chronic Pain in US Veterans Treated Within the Veterans Health Administration: Implications for Expansion in US Healthcare Systems

**DOI:** 10.1007/s11606-021-07370-8

**Published:** 2022-01-19

**Authors:** Zachary L. Mannes, Malki Stohl, David S. Fink, Mark Olfson, Katherine M. Keyes, Silvia S. Martins, Jaimie L. Gradus, Andrew J. Saxon, Charles Maynard, Ofir Livne, Sarah Gutkind, Deborah S. Hasin

**Affiliations:** 1 Department of Epidemiology, Columbia University Mailman School of Public Health, New York, NY USA; 2 New York State Psychiatric Institute, 1051 Riverside Drive, Box 123, New York, NY 10032 USA; 3 Department of Psychiatry, Columbia University Irving Medical Center, New York, NY USA; 4 Department of Epidemiology, Boston University School of Public Health, Boston, MA USA; 5 Department of Psychiatry, Boston University School of Medicine, Boston, MA USA; 6 Department of Psychiatry & Behavioral Sciences, University of Washington School of Medicine, Seattle, WA USA; 7 VA Puget Sound Healthcare System, Seattle, WA USA

**Keywords:** chronic pain, prescription opioid use, non-pharmacological pain treatment, military veterans, Veterans Health Administration

## Abstract

**Background:**

Consensus guidelines recommend multimodal chronic pain treatment with increased use of non-pharmacological treatment modalities (NPM), including as first-line therapies. However, with many barriers to NPM uptake in US healthcare systems, NPM use may vary across medical care settings. Military veterans are disproportionately affected by chronic pain. Many veterans receive treatment through the Veterans Health Administration (VHA), an integrated healthcare system in which specific policies promote NPM use.

**Objective:**

To examine whether veterans with chronic pain who utilize VHA healthcare were more likely to use NPM than veterans who do not utilize VHA healthcare.

**Design:**

Cross-sectional nationally representative study.

**Participants:**

US military veterans (*N* = 2,836).

**Main Measures:**

In the 2019 National Health Interview Survey, veterans were assessed for VHA treatment, chronic pain (i.e., past 3-month daily or almost daily pain), symptoms of depression and anxiety, substance use, and NPM (i.e., physical therapy, chiropractic/spinal manipulation, massage, psychotherapy, educational class/workshop, peer support groups, or yoga/tai chi).

**Key Results:**

Chronic pain (45.2% vs. 26.8%) and NPM use (49.8% vs. 39.4%) were more prevalent among VHA patients than non-VHA veterans. After adjusting for sociodemographic characteristics, psychiatric symptoms, physical health indicators, and use of cigarettes or prescription opioids, VHA patients were more likely than non-VHA veterans to use any NPM (adjusted odds ratio [aOR] = 1.52, 95% CI: 1.07–2.16) and multimodal NPM (aOR = 1.80, 95% CI: 1.12–2.87) than no NPM. Among veterans with chronic pain, VHA patients were more likely to use chiropractic care (aOR = 1.90, 95% CI = 1.12–3.22), educational class/workshop (aOR = 3.02, 95% CI = 1.35–6.73), or psychotherapy (aOR = 4.28, 95% CI = 1.69–10.87).

**Conclusions:**

Among veterans with chronic pain, past-year VHA use was associated with greater likelihood of receiving NPM. These findings may suggest that the VHA is an important resource and possible facilitator of NPM. VHA policies may offer guidance for expanding use of NPM in other integrated US healthcare systems.

## INTRODUCTION

Chronic pain, i.e., pain that persists or recurs for 3 months or more^1, 2^, affects 20% of US adults,^3^ contributes to an annual economic cost of $635 billion^4^, and is associated with disability, cognitive impairment, cardiovascular disease, and psychiatric and substance use disorders.^5–8^ Opioid prescribing was previously considered standard of care for chronic pain,^9^ and the national opioid dispensing rate increased from 1999 to 2012, accompanied by increases in morbidity and mortality.^10, 11^ After the risks of excessive prescription of opioids became widely recognized, rates of opioid prescribing declined^12^ and the Centers for Disease Control and Prevention (CDC) began recommending the use of non-pharmacological modalities (NPM) such as physical rehabilitation, chiropractic care, and psychological treatments as first-line alternatives to opioids for chronic pain.^13^ Use of NPM, particularly multimodal treatment (i.e., concurrent use of ≥ 2 NPM), can yield improvements in pain, functioning, and quality of life,^14, 15^ is associated with lower healthcare costs,^16^ and may reduce receipt of prescription opioids.^17^ However, high cost, limited provider reimbursement, and poor access, or the inability to obtain affordable, convenient healthcare services,^18^ has limited use of NPM within US healthcare settings.^19–23^ With decreasing rates of opioid prescribing in the US,^12^ patients with chronic pain may be left untreated unless alternative therapies are provided. Therefore, identifying healthcare settings where patients are likely to use NPM, or those where NPM resources are less available, may guide initiatives aimed at improving access to these treatments, particularly among populations vulnerable to chronic pain.

Chronic pain is particularly prevalent among US military veterans, affecting approximately one-third of veterans receiving healthcare through the Veterans Health Administration (VHA).^24, 25^ Opioid prescribing in the VHA increased from 17 to 24% between 2001 and 2009,^26^ and was associated with opioid-related hospitalizations, overdose, and self-inflicted injuries.^27, 28^ These harms were highest among veterans with depression and anxiety disorders,^28^ which are more prevalent in veterans than the US general population.^29^ To mitigate these consequences, the VHA enacted the 2013 Opioid Safety Initiative, a multimodal clinical program that contributed to a 64% decrease in opioid prescribing.^30, 31^ Consistent with CDC guidelines, the VHA now emphasizes use of NPM,^32^ which is associated with lower risk of overdose, suicidal ideation, and self-injurious behavior.^33^

The VHA is the largest integrated healthcare system in the US, providing healthcare to over 5.5 million veterans annually, including chronic pain services without the need for outside referrals or prior authorizations.^34^ The VHA also promotes a holistic approach to chronic pain treatment^35, 36^ through the Whole Health Program, a wellness initiative leveraging complimentary and integrative health services.^37^ However, not all veterans are eligible for VHA services due to not meeting eligibility requirements such as low income or service-related disabilities, and 17% of veterans who are enrolled in the VHA use it as their only source of care.^38, 39^ Consequently, use of NPM may depend on whether veterans receive their care in the VHA, where some barriers remain although concerted efforts are in place to overcome them,^19, 20, 40, 41^ or in other healthcare systems with persisting obstacles to NPM provision.^21–23^ In this study, we investigated whether veterans with chronic pain who received their care within the VHA’s integrated system were more or less likely to use NPM than veterans outside the VHA. Drawing on data from the 2019 National Health Interview Survey (NHIS), we first examined differences in the prevalence of chronic pain between veterans receiving their healthcare from the VHA and those who do not, as these findings may have important implications for NPM resource allocation. Second, we examined associations between VHA healthcare use and NPM among veterans with chronic pain. We hypothesized that among veterans with chronic pain, VHA patients would be more likely to use NPM than non-VHA veterans.

## METHODS

### Sample and Design

Data were derived from the 2019 NHIS Adult component (https://www.cdc.gov/nchs/nhis/2019nhis.htm;*N* = 31,997), a yearly health survey that characterizes the self-reported physical and mental health of the non-institutionalized civilian population of the US, including military veterans. Trained interviewers collected NHIS data from January to December, 2019. The 2019 NHIS adult response rate was 59.1%. The analytic sample for this report consisted of participants who self-reported a history of military service and at least one healthcare visit within the prior 12 months (*N* = 2,836), of whom 10 were excluded due to missing age information, yielding an analytic sample of 2,826 respondents. The New York State Psychiatric Institute’s Institutional Review Board did not require human subjects review as we analyzed de-identified, public-use data.

### Measures

#### Chronic Pain andHigh-Impact Chronic Pain

Participants were asked, “*In the past three months, how often did you have pain? Would you say never, some days, most days, or every day*?” We defined chronic pain as pain “most days” or “every day”.^1, 2^ Participants were then asked, “*Over the past three months, how often did your pain limit your life or work activities? Would you say never, some days, most days, or every day*?” High-impact chronic pain was defined as pain that limited life or work activities most days or every day. These definitions have been used in other NHIS studies^3, 42–45^ and to assess pain among veterans.^24–26^

#### Exposure

##### VHA Healthcare

Most VHA patients utilize care at VHA facilities, although the VHA also covers care by designated community clinicians when it cannot provide needed care.^38^ Veterans were asked: “*During the past 12 months, did you receive any care at a Veteran*’*s Health Administration facility or receive any other health care paid for by the VA?*” Interviewers prompted participants that VHA care included VA hospitals, medical centers, outpatient clinics, and nursing homes. A dichotomous variable assessed past 12-month use of VHA healthcare (labeled hereafter as “VHA patients” and “non-VHA veterans).”

#### Outcome

##### NPM

The VHA recommends several evidence-based NPM.^46^ Participants were asked about their NPM use for pain with the follow question: “Over the past three months, did you use any of the following to manage your pain?” NPM included physical therapies (physical, rehabilitative, or occupational therapy); chiropractic/spinal manipulation; massage; psychotherapy; educational class/workshop; peer support groups; yoga/tai chi; or meditation/guided imagery/other relaxation techniques. Participants were not asked where they obtained these services, but they could endorse use of multiple NPM. Dichotomous variables were created for each treatment modality (use; no use). We also created variables indicating use of ≥ 1 NPM and ≥ 2 NPM, since multimodal pain treatment is associated with improved health outcomes.^14, 15^

#### Covariates

##### Prescription Opioid Use

Participants were asked: “*During the past 3 months, did you take a prescription opioid to treat long-term or chronic pain, such as low back pain or neck pain, frequent headaches or migraines, or joint pain or arthritis*?” A dichotomous (yes; no) variable indicated past 3-month prescription opioid use for chronic pain.

##### Depressive Symptoms

The Patient Health Questionnaire (PHQ-8) assessed depression symptoms over the past 2 weeks with eight Likert scale items ranging from “0” (not at all) to “3” (nearly every day). Scores were dichotomized into no/mild symptoms (score 0–9), and moderate/severe symptoms (score ≥ 10).^47^ The PHQ-8 has strong sensitivity and specificity for major depressive disorder.^47^

##### Anxiety Symptoms

The Generalized Anxiety Disorder 7-item Assessment (GAD-7) measured anxiety over the past 2 weeks with seven Likert scale items ranging from “0” (not at all) to “3” (nearly every day). Participants were dichotomized into none/mild symptoms (score 0–9), and moderate/severe symptoms (score ≥ 10).^48^ The GAD-7 has high internal and external reliability.^48^

##### Cigarette Use

Current smokers (past 30-day use), former smokers (use more than 30 days ago), and never smokers were categorized into three, mutually exclusive groups. Cigarette use is associated with chronic pain^49^ and healthcare utilization^50^ among VHA patients.

##### Body Mass Index (BMI)

BMI was calculated using self-reported height and weight. Due to low prevalence of underweight in this sample (1.61%), participants were categorized into two groups: underweight and healthy weight (BMI < 24.9) vs. overweight or obese (BMI ≥ 25). Overweight and obesity are associated with chronic pain and other morbidities contributing to use of healthcare services.^51^

##### Sociodemographic Characteristics

Age (18–34, 35–49, 50–64, ≥ 65), sex (male, female), race/ethnicity (non-Hispanic/White, non-Hispanic/Black, non-Hispanic/Asian, Hispanic/Latino, other), education (high school or equivalent, college or more), family income relative to the federal poverty line (FPL; < 100% FPL, 100–199%, 200–399% FPL, ≥ 400% FPL), US region of residence (Northeast, Midwest, South, West), and county urbanicity (large central metropolitan, large fringe metropolitan, medium/small metropolitan, nonmetropolitan).^52^

### Statistical Analysis

Analyses were conducted using SAS-callable SUDAAN 11.0.1 and were weighted and adjusted for the complex survey design. Among all veterans, chi-square tests assessed differences in prevalence of sociodemographic characteristics, psychiatric symptoms, cigarette use, chronic pain, and high-impact chronic pain by VHA utilizer status. Among veterans with chronic pain, chi-square tests assessed differences in past 3-month use of prescription opioids or NPM among VHA and non-VHA veterans. Binary logistic regression models were then run to examine associations between VHA utilization and use of any NPM (≥ 1 vs. 0) or use of each NPM (yes vs. no) adjusting for age, sex, race/ethnicity, education, income, US region, urbanicity, BMI, generalized anxiety or depressive symptoms, high-impact chronic pain, and use of cigarettes or prescription opioids. A multinomial logistic regression model was also fit to examine the association between VHA utilization with a three-level NPM use outcome (1 NPM, ≥ 2 NPM, vs. 0) adjusting for covariates, from which adjusted odds ratios (aOR) with 95% confidence intervals (CIs) were derived.

## RESULTS

### Sample Characteristics

Veterans were primarily male (89.1%), non-Hispanic, White (78.7%), with at least some college education (69.2%), and many were aged 65 or older (50.3%), had family income ≥ 400% FPL (45.5%), and resided in metropolitan areas (82.3%). Approximately 35% of veterans used the VHA within the past 12 months. Nearly 33% of veterans had chronic pain, and 19.1% had high-impact chronic pain.

Compared to non-VHA veterans, VHA patients were more likely to be racial/ethnic minorities (38.3% vs. 18.7%), have lower income (64.4% vs. 49.6%), and have moderate/severe symptoms of GAD (10.1% vs. 3.4%) or MDD (12.4% vs. 6.1%). VHA patients were also more likely than non-VHA veterans to have chronic pain (45.2% vs. 26.8%) and high-impact chronic pain (26.0% vs. 15.2%; Table 1).

**Table 1:** Characteristics of study sample (*N* = 2,836)

Variable	VHA patients (*N* = 983)	Non-VHA veterans (*N* = 1,853)	All veterans (*N* = 2,836)	
	% (SE)	% (SE)	% (SE)	*p* value
**Age**				0.32
18–34	11.25 (1.41)	8.59 (1.01)	9.47 (0.85)	
35–44	9.33 (1.08)	8.54 (0.81)	8.80 (0.68)	
45–54	11.54 (1.36)	14.09 (1.07)	13.25 (0.84)	
55–64	17.32 (1.36)	18.61 (1.06)	18.19 (0.82)	
≥ 65	50.55 (2.02)	50.17 (1.40)	50.30 (1.17)	
**Sex**				0.68
Men	89.55 (1.22)	88.91 (0.87)	89.12 (0.67)	
Women	10.45 (1.22)	11.09 (0.87)	10.88 (0.67)	
**Race/ethnicity**				**< 0.01**
Non-Hispanic, White	70.86 (1.99)	82.59 (1.25)	78.73 (1.13)	
Non-Hispanic, Black	18.05 (1.67)	8.79 (0.94)	11.84 (0.86)	
Non-Hispanic, Asian	2.39 (0.80)	1.30 (0.33)	1.66 (0.34)	
Hispanic	6.56 (0.88)	4.66 (0.68)	5.28 (0.53)	
Other ^a^	2.14 (0.50)	2.67 (0.48)	2.49 (0.38)	
**Education**				0.33
High school	32.25 (1.88)	30.05 (1.36)	30.77 (1.14)	
Some college or more	67.75 (1.88)	69.95 (1.36)	69.23 (1.14)	
**Family income (FPL)**				**< 0.001**
< 100%	6.07 (0.92)	4.07 (0.56)	4.73 (0.49)	
100–199%	19.36 (1.43)	11.82 (0.97)	14.30 (0.79)	
200–399%	38.98 (1.87)	33.71 (1.30)	35.45 (1.06)	
≥ 400%	35.58 (1.82)	50.40 (1.47)	45.52 (1.20)	
**US region**				0.44
Northeast	14.83 (1.79)	17.29 (1.12)	16.48 (1.04)	
Midwest	21.00 (1.59)	21.05 (1.31)	21.03 (1.09)	
South	44.61 (2.11)	41.29 (1.66)	42.38 (1.44)	
West	19.57 (1.71)	20.37 (1.28)	20.11 (1.12)	
**Urbanicity**^**b**^				0.14
Metropolitan	80.59 (1.65)	83.21 (1.19)	82.34 (1.06)	
**GAD symptoms**^**c**^				**< 0.01**
Moderate/severe	10.16 (1.09)	3.40 (0.51)	5.63 (0.49)	
**MDD symptoms**^**d**^				**< 0.01**
Moderate/severe	12.46 (1.26)	6.10 (0.81)	8.20 (0.68)	
**Cigarette use**^**e**^				0.08
Current smoker	17.76 (1.40)	14.68 (1.16)	15.69 (0.91)	
**BMI**^**f**^				0.23
Overweight or obese	77.22 (1.50)	74.92 (1.24)	75.68 (0.99)	
**Chronic pain**^**g**^	45.26 (1.90)	26.88 (1.21)	32.93 (1.07)	**< 0.01**
**High-impact** **chronic pain**^**h**^	26.04 (1.99)	15.20 (1.24)	19.15 (1.10)	**< 0.01**

Note. Bold values indicate significance at *p* < 0.05. *VHA* Veterans Health Administration, *FPL* federal poverty line, *GAD* generalized anxiety disorder, *MDD* major depressive disorder, *BMI* body mass index

^a^Included biracial adults

^b^Metropolitan defined as large central, fringe, medium, or small metropolitan vs. non-metropolitan

^c^Generalized Anxiety Disorder Scale-7 item (GAD-7) assessed generalized anxiety disorder symptoms during the past 2 weeks. Participants were categorized into none to mild (0–9) symptoms and moderate to severe symptoms (10–21)

^d^Patient Health Questionnaire Depression Scale-8 item (PHQ-8) assessed depressive symptoms during the past 2 weeks. Participants were categorized into none to mild (0–9) and moderate to severe (15–24) symptom severity

^e^Includes combustible or electronic cigarette use

^f^Overweight or obese vs. underweight or healthy weight

^g^Pain every day or most days during the past 3 months

^h^Chronic pain that interferes with daily activities every day or on most days during the past 3 months

### Pain Treatment

Among veterans with chronic pain, approximately 14.9% used prescription opioids, 43.8% used any NPM, and 16.4% used multimodal NPM. The most common NPM for pain were physical therapies (21.1%), massage (15.3%), relaxation strategies (13.3%), and chiropractic care (11.4%). VHA patients were more likely than non-VHA veterans to use any NPM (49.8% vs. 39.4%), or multimodal NPM (23.2% vs. 16.4%). Compared to non-VHA veterans, VHA patients were also more likely to use physical therapies (24.4% vs. 18.3%), chiropractic care (14.1% vs. 9.3%), an educational class/workshop (8.3% vs. 3.5%), meditation/relaxation strategies (16.0% vs. 11.0%), yoga/tai chi (7.2% vs. 4.9%), and psychotherapy (7.1% vs. 1.5%; Table 2).

**Table 2:** Prevalence of pain treatment modalities among veterans with chronic pain (*N* = 936)^a^

Variable	VHA patients (*N* = 445)	Non-VHA veterans (*N* = 491)	All veterans (*N* = 936)	
	% (SE)	% (SE)	% (SE)	*p* value
Opioid use ^b^	13.84 (1.71)	15.86 (2.17)	14.94 (1.39)	0.479
NPM ^c^				
Physical therapies	24.48 (2.46)	18.30 (1.93)	21.10 (1.55)	**0.04**
Chiropractic care	14.11 (1.91)	9.32 (1.65)	11.49 (1.23)	0.06
Massage	15.42 (2.06)	15.29 (2.16)	15.34 (1.43)	0.96
Psychotherapy	7.13 (1.65)	1.59 (0.59)	4.09 (0.82)	**< 0.01**
Education class/workshop	8.36 (1.54)	3.54 (0.89)	5.72 (0.85)	**< 0.01**
Peer support group	2.65 (1.06)	0.78 (0.36)	1.63 (0.52)	0.09
Yoga/tai chi	7.24 (1.32)	4.99 (1.31)	6.01 (1.03)	0.17
Meditation/relaxation techniques	16.05 (2.02)	11.09 (1.69)	13.34 (1.29)	0.06
NPM				
Yes (≥ 1)	49.83 (2.82)	39.48 (2.66)	43.80 (2.01)	**< 0.01**
Total number of NPM				**0.01**
0	50.17 (2.82)	60.52 (2.66)	55.84 (2.01)	
1	26.59 (2.64)	23.02 (2.23)	24.63 (1.76)	
≥ 2	23.23 (2.41)	16.46 (2.14)	19.53 (1.61)	

Note. Bold values indicate significance at *p* < 0.05. *VHA* Veterans Health Administration, *NPM*non-pharmacological treatment modalities

^a^Includes 936 participants reporting daily or almost daily pain over the past 3 months

^b^Past 3-month prescription opioid use for chronic pain

^c^Past 3-month pain treatment (yes, no)

### Adjusted Associations Between VHA Utilization and NPM

**Figure 1:**
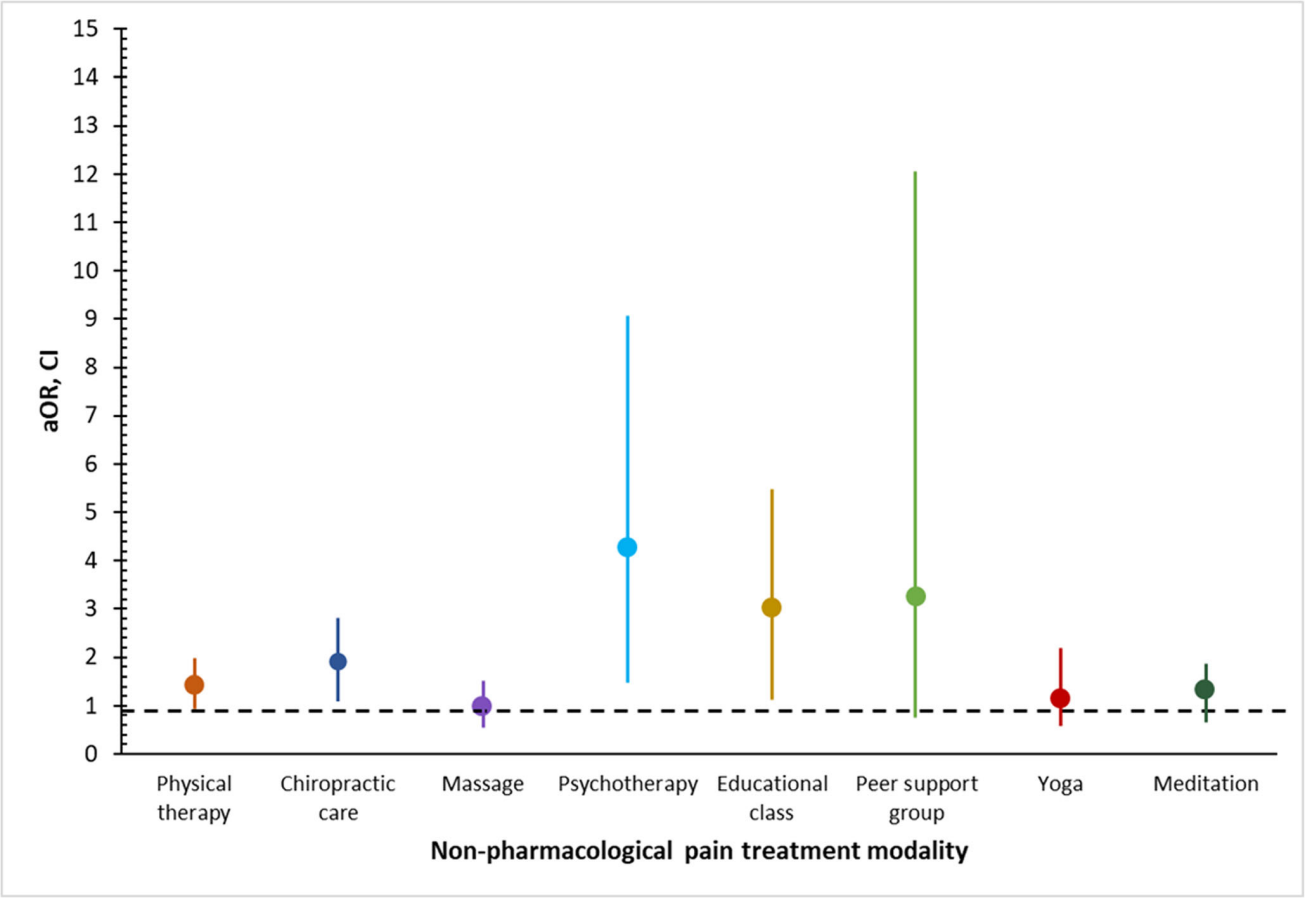
Adjusted odds ratios (aOR) for associations of VHA use and each NPM. Note. Lines correspond to 95% CI around the aOR. Bars not overlapping with 1.00 indicate significant association. VHA, Veterans Health Administration; NPM, non-pharmacological treatment modalities. Models adjusted for age, sex, race/ethnicity, education, family income, US region, urbanicity, generalized anxiety disorder symptoms, major depressive disorder symptoms, BMI, and use of cigarettes or prescription opioids.

Compared to non-VHA veterans with chronic pain, VHA patients were more likely to use any NPM (aOR = 1.52, 95% CI: 1.07–2.16; Table 3). VHA veterans were also more likely than non-VHA veterans to use multimodal NPM (aOR = 1.80, 95% CI: 1.12–2.87) than no NPM (Table 4). Among veterans with chronic pain, VHA veterans were more likely to use chiropractic care (aOR = 1.90, 95% CI: 1.12–3.22), educational class/workshop (aOR = 3.02, 95% CI: 1.35–6.73), or psychotherapy (aOR = 4.28, 95% CI: 1.69–10.87; Fig. 1).

**Table 3:** Association of VHA utilization and any NPM use among veterans with chronic pain (*N* = 936)

Variable	≥ 1 NPM (*n* = 410) (vs. no NPM)
	AOR^a^ (95% CI)
**VHA utilizer status**	
Non-VHA veteran	ref
VHA patient	**1.52 (1.07, 2.16)**
**Age**	
18–34	ref
35–44	0.77 (0.31, 1.90)
45–54	0.46 (0.21, 1.01)
55–64	**0.36 (0.17, 0.78)**
≥ 65	**0.26 (0.13, 0.54)**
**Sex**	
Male	ref
Female	1.60 (0.94, 2.72)
**Race/ethnicity**	
Non-Hispanic, White	ref
Non-Hispanic, Black	0.70 (0.38, 1.29)
Non-Hispanic, Asian	0.80 (0.14, 4.54)
Hispanic	1.00 (0.36, 2.78)
Other^b^	1.25 (0.56, 2.78)
**Education**	
High school	ref
Some college or more	**2.07 (1.42, 3.01)**
**Family income**	
< 100% FPL	ref
100–199% FPL	1.00 (0.47, 2.13)
200–399% FPL	1.29 (0.68, 2.44)
≥ 400% FPL	1.70 (0.88, 3.30)
**US region**	
Northeast	ref
Midwest	1.00 (0.55, 1.79)
South	0.86 (0.51, 1.46)
West	1.09 (0.63, 1.90)
**GAD symptoms**^**c**^	
None/mild	ref
Moderate/severe	1.41 (0.65, 3.07)
**MDD symptoms**^**d**^	
None/mild	ref
Moderate/severe	0.84 (0.42, 1.71)
**Cigarette use**^**e**^	
Never	ref
Former	0.85 (0.57, 1.28)
Current	**0.58 (0.36, 0.94)**
**Opioid use**^**f**^	
No	ref
Yes	1.60 (0.97, 2.65)
**High-impact chronic pain**^**g**^	
No	ref
Yes	**1.60 (1.13, 2.25)**
**BMI**	
Underweight or normal weight	ref
Overweight or obese	0.74 (0.48,1.14)
**Urbanicity**^**h**^	
Nonmetropolitan	ref
Metropolitan	1.26 (0.82, 1.91)

Note. Bold values indicate *p* < 0.05. *aOR* adjusted odds ratio, *NPM*non-pharmacological treatment modalities, *VHA* Veterans Health Administration, *FPL* federal poverty line, *GAD* generalized anxiety disorder, *MDD* major depressive disorder, *BMI* body mass index

^a^Model adjusted for age, sex, race/ethnicity, education, family income, US region, GAD, MDD, and use of cigarettes or prescription opioids, high-impact chronic pain, BMI, and urbanicity

^b^Included biracial adults

^c^Generalized Anxiety Disorder Scale-7 item (GAD-7) assessed generalized anxiety disorder symptoms during the past 2 weeks. Participants were categorized into none to mild (0–9) symptoms and moderate to severe symptoms (10–21)

^d^Patient Health Questionnaire Depression Scale-8 item (PHQ-8) assessed depressive symptoms during the past 2 weeks. Participants were categorized into none to mild (0–9) and moderate to severe (15–24) symptom severity

^e^Includes combustible or electronic cigarette use

^f^Past 3-month prescription opioid use for chronic pain

^g^Chronic pain that interferes with daily activities every day or on most days during the past 3 months

^h^Metropolitan defined as large central, fringe, medium, or small metropolitan

**Table 4:** Association of VHA utilization with use of 1 or ≥ 2 NPM among veterans with chronic pain (*N* = 936)

Variable	1 NPM (vs. no NPM; *n* = 231)	≥ 2 NPM (vs. no NPM; *n* = 179)
	AOR^a^ (95% CI)	AOR^a^ (95% CI)
**VHA utilizer status**		
Non-VHA veteran	ref	ref
VHA patient	1.36 (0.91, 2.03)	**1.80 (1.12, 2.87)**
Age		
18–34	ref	ref
35–44	0.91 (0.28, 3.01)	0.70 (0.25, 2.01)
45–54	0.68 (0.23, 2.00)	**0.31 (0.12, 0.80)**
55–64	0.52 (0.18, 1.50)	**0.24 (0.10, 0.60)**
≥ 65	0.41 (0.15, 1.15)	**0.16 (0.07, 0.37)**
**Sex**		
Male	ref	ref
Female	1.08 (0.55, 2.14)	**2.21 (1.17, 4.19)**
**Race/ethnicity**		
Non-Hispanic, White	ref	ref
Non-Hispanic, Black	0.80 (0.41, 1.59)	0.56 (0.25, 1.26)
Non-Hispanic, Asian	0.65 (0.09, 4.73)	0.81 (0.11, 5.81)
Hispanic	1.43 (0.48, 4.27)	0.62 (0.18, 2.09)
Other^b^	0.83 (0.32, 2.18)	1.79 (0.67, 4.79)
**Education**		
High school	ref	ref
Some college or more	**1.95 (1.23, 3.09)**	**2.30 (1.41, 3.77)**
**Family income**		
< 100% FPL	ref	ref
100–199% FPL	1.06 (0.41, 2.73)	0.92 (0.35, 2.40)
200–399% FPL	1.43 (0.61, 3.35)	1.10 (0.46, 2.59)
≥ 400% FPL	1.57 (0.66, 3.77)	1.91 (0.84, 4.34)
**US region**		
Northeast	ref	ref
Midwest	1.01 (0.51, 1.99)	0.98 (0.44,2.17)
South	0.85 (0.46,1.56)	0.90 (0.45,1.80)
West	0.81 (0.43, 1.53)	1.61 (0.79,3.27)
**GAD symptoms**^**c**^		
None/mild	ref	ref
Moderate/severe	1.81 (0.75, 4.36)	1.09 (0.44, 2.73)
**MDD symptoms**^**d**^		
None/mild	ref	ref
Moderate/severe	0.52 (0.25, 1.11)	1.44 (0.61, 3.41)
**Cigarette use**^**e**^		
Never	ref	ref
Former	1.04 (0.66, 1.65)	0.64 (0.38, 1.06)
Current	0.70 (0.40, 1.21)	**0.43 (0.22, 0.83)**
**Opioid use**^**f**^		
No	ref	ref
Yes	1.46 (0.85, 2.52)	1.81 (0.93, 3.53)
**High-impact chronic pain**^**g**^		
No	ref	ref
Yes	1.40 (0.94, 2.09)	**1.95 (1.22, 2.13)**
**BMI**		
Underweight or normal weight	ref	ref
Overweight or obese	0.73 (0.45,1.20)	0.78 (0.45,1.37)
**Urbanicity**^**h**^		
Nonmetropolitan	ref	ref
Metropolitan	1.28 (0.81, 2.02)	1.22 (0.64, 2.33)

Note. Bold values indicate *p* < 0.05. *aOR* adjusted odds ratio, *NPM*non-pharmacological treatment modalities, *VHA* Veterans Health Administration, *FPL* federal poverty line, *GAD* generalized anxiety disorder, *MDD* major depressive disorder, *BMI* body mass index

^a^Model adjusted for age, sex, race/ethnicity, education, family income, US region, GAD, MDD, and use of cigarettes or prescription opioids, high-impact chronic pain, BMI, and urbanicity

^b^Included biracial adults

^c^Generalized Anxiety Disorder Scale-7 item (GAD-7) assessed generalized anxiety disorder symptoms during the past 2 weeks. Participants were categorized into none to mild (0–9) symptoms and moderate to severe symptoms (10–21)

^d^Patient Health Questionnaire Depression Scale-8 item (PHQ-8) assessed depressive symptoms during the past 2 weeks. Participants were categorized into none to mild (0–9) and moderate to severe (15–24) symptom severity

^e^Includes combustible or electronic cigarette use

^f^Past 3-month prescription opioid use for chronic pain

^g^Chronic pain that interferes with daily activities every day or on most days during the past 3 months

^h^Metropolitan defined as large central, fringe, medium, or small metropolitan

## DISCUSSION

In a nationally representative survey of the US population, we examined differences in chronic pain and NPM use among military veterans by their use of VHA healthcare. In this study, VHA patients demonstrated greater prevalence of chronic pain and moderate-severe symptoms of anxiety and depression compared to non-VHA veterans. Importantly, among veterans with chronic pain, VHA patients were also more likely than non-VHA veterans to use NPM, including chiropractic care, psychotherapy, an educational class/workshop, or multimodal pain treatment. These results suggest that the VHA is an important resource and possible facilitator of NPM. These findings may help inform healthcare delivery practices of non-VHA healthcare systems that are facing challenges incorporating evidence-based NPM into routine care.

Over 45% of VHA patients had chronic pain and 25% had high-impact chronic pain. These rates are even higher than those found in previous national studies, possibly due to the aging of former combat veterans susceptible to chronic pain^53^ or increasing national rates of chronic pain^43^ and the comorbidities that exacerbate it.^54^ Furthermore, VHA patients were disproportionately affected by psychiatric symptoms and cigarette use. The burden caused by high-impact chronic pain and psychiatric symptoms among VHA patients could explain their greater use of NPM, as this group may be seeking treatment for these morbidities. Epidemiological research demonstrates a bi-directional association between chronic pain, and psychiatric or substance use disorders,^8^ though reduction in pain intensity with psychosocial interventions can reduce the severity of substance use and mental health symptoms.^55, 56^ NPM offer treatment for the physical (e.g., strength and flexibility), psychological (e.g., self-efficacy and management of catastrophizing), and social (e.g., social isolation and unemployment) consequences of chronic pain that perpetuate its health comorbidities among VHA patients.^57^ Therefore, continued attempts to expand NPM in among VHA patients may assist with reducing their high physical and psychiatric burden.

In this study, NPM use was more common among VHA patients, a group with significantly fewer economic resources than non-VHA veterans. Among non-VHA patients, insurance limits for NPM are common,^21^ and therapies such as acupuncture, massage, and mind-body interventions (i.e., yoga and tai chi) are not routinely covered, while psychotherapies for pain conditions are not reimbursable in most states.^22^ Despite an almost universal lack of insurance coverage for many NPM in the US, expansion of coverage for these therapies increases NPM use^58^ and provides long-term cost-saving benefits.^59, 60^ Further empirical support for the financial and health benefits of NPM expansion might help overcome existing reimbursement constraints. In addition, transportation difficulties and greater distance to clinics that offer NPM may limit use of these services.^19, 20^ Use of telemedicine in VHA and non-VHA health systems has increased due to the COVID-19 pandemic^61, 62^ and may be useful for overcoming challenges in providing NPM, particularly for patients in rural areas where NPM access is limited by provider shortages, appointment availability, and number of specialty pain clinics.^63^ In this sample, women, younger adults, and veterans with higher educational attainment were also more likely to use NPM. These findings are like those of a prior VHA study,^64^ underscoring the need to expand NPM to sociodemographic groups less likely to use these services.

Treatment preferences, provider beliefs about pain treatment, and VHA efforts to improve access to NPM may explain the higher rates of these services among VHA patients. Historically, VHA patients were unenthusiastic about NPM.^19^ However, as prescribed opioids for chronic pain management are now discouraged, patient interest in NPM may be increasing.^41, 65^ VHA providers now also prefer psychotherapy and physical therapy interventions for chronic pain.^66^VHA-implemented clinical and research initiatives have likely also expanded NPM use among veterans with chronic pain. For example, the VHA National Pain Management Strategy has advanced a biopsychosocial approach to chronic pain treatment.^36^ This program has informed the Pain Management Collaboratory, an $88 million initiative of the NIH and VA/DoD that has reinforced NPM research and clinical implementation.^35^ The VHA Whole Health Program has also increased dissemination of NPM,^65^ and access to NPM may further improve under the Maintaining Internal Systems and Strengthening Integrated Outside Networks (MISSION) Act.^67, 68^

Study limitations are noted. First, because the 2019 NHIS was the first version to assess NPM, data were cross-sectional, and we cannot determine the temporality of the associations. Second, our measure of NPM was limited to use within the past 3 months, and we were unable to examine frequency or duration of NPM use or whether VHA patients received NPM from non-VHA providers. Third, chronic pain was determined via self-report and NHIS did not have numerical ratings of pain intensity. However, our definition of chronic pain was consistent with the definition provided by the International Association for the Study of Pain,^1, 2^ and high-impact chronic pain was used to approximate pain severity. Fourth, symptoms of posttraumatic stress disorder, use of alcohol or illicit substances, and non-opioid medications for pain (e.g., nonsteroidal anti-inflammatory drugs [NSAIDS], gabapentinoids, serotonin and norepinephrine reuptake inhibitors [SNRIs]) were not assessed in the 2019 NHIS, and therefore no conclusions can be drawn regarding their association with NPM. Fifth, this was a sample of veterans, who are largely white males of middle age or older, many of whom have service-connected medical disabilities. Of these, VHA patients receive pain services regardless of insurance type (e.g., CHAMPVA, TRICARE) and may qualify for subsidized services despite limited income. Therefore, our findings may not be generalizable to other patient populations, including those that experience insurance constraints for NPM. Despite these limitations, this investigation had notable strengths. This study had a sufficient sample size to control for many health and sociodemographic confounders.^69^ Moreover, the 2019 NHIS is a rich data source to examine many NPM. Finally, we examined the prevalence of chronic pain and NPM between VHA patients and non-VHA veterans, which is an important advance over most pain studies of veterans that often include only VHA patients.

Studies examining access to NPM for chronic pain are important to inform clinical practice, particularly as prescriptions for opioids wane while chronic pain patients continue to require care. Our study demonstrated that VHA patients, a population highly burdened by chronic pain and its comorbidities, were more likely than non-VHA veterans to use psychotherapy, chiropractic care, educational classes/workshops, or multimodal NPM. This study supports previous findings suggesting that VHA initiatives have expanded use of NPM.^40^ Future studies should assess longitudinal changes in NPM since enactment of the CDC and VA/DoD opioid prescribing guidelines in 2016–2017, and the influence of the COVID-19 pandemic on NPM use. Furthermore, implementation research is needed to determine how NPM can best be disseminated in other health systems. Perhaps most importantly, future research should aim to understand how the shift away from prescription opioids towards NPM has affected population health and well-being, e.g., pain, morbidity, and mortality. Studies are needed to ascertain rates of NPM use among VHA patients deprescribed long-term prescription opioids, and whether use of NPM can offer health benefits to existing opioid taper protocols or after tapers have ended. Such research has important health implications for patients susceptible to the consequences of untreated chronic pain and opioid discontinuation.^70, 71^
